# Re-delimitation of the genus *Emertonella* (Araneae, Theridiidae, Hadrotarsinae) and taxonomic notes on *Euryopis* and *Phycosoma* from China

**DOI:** 10.3897/zookeys.1270.175743

**Published:** 2026-02-26

**Authors:** Changhao Hu, He Zhang, Minjia Jiang, Fengjie Liu

**Affiliations:** 1 College of Life Sciences, Hunan Normal University, Changsha 410081, China Faculty of Resources and Environmental Sciences, Hubei University Wuhan China; 2 Arachnid Resource Centre of Hubei & Hubei Key Laboratory of Regional Development and Environmental Response, Faculty of Resources and Environmental Sciences, Hubei University, Wuhan 430062, China School of Life Sciences, Hubei University Wuhan China; 3 Centre for Behavioral Ecology and Evolution, School of Life Sciences, Hubei University, Wuhan 430062, China Hunan Normal University Changsha China; 4 College of Physics and Electronic Engineering, Xingtai University, Xingtai, Hebei 054001, China Xingtai University Xingtai China; 5 Jiujiang Forestry Science Research Institute, Jiujiang, Jiangxi 332006, China Jiujiang Forestry Science Research Institute Jiujiang China

**Keywords:** Cobweb spiders, morphology, new combinations, redescription, taxonomy

## Abstract

Hadrotarsinae Thorell, 1881 is a subfamily of cobweb spiders (Theridiidae) distributed worldwide and generally regarded as specialized ant predators. The taxonomy of Hadrotarsinae is problematic due to unclear delimitation of genera, leading to likely misplacement of many species. In the current paper, we provide taxonomic amendments for *Emertonella* Bryant, 1945, *Euryopis* Menge, 1868, and *Phycosoma* O. Pickard-Cambridge, 1880 based on Chinese materials. Examination of *Eu. deplanata* Schenkel, 1936 and *Em. taczanowskii* (Keyserling, 1886) supports Yoshida’s concept of *Emertonella* and provides additional morphological evidence for refining the generic diagnosis. Accordingly, *Eu. deplanata* is formally transferred, with 30 additional species, from *Euryopis* to *Emertonella*, and *Em. taczanowskii* is redescribed. Furthermore, *Physcoa mingyaoi* (Yin, 2012), **comb. nov**. ex. *Euryopis* and *Lasaeola stigmosum* (Yin, 2012), **comb. nov**. ex. *Phycosoma* are transferred, and one new synonym is proposed, *Phycosoma amamiense* (Yoshida, 1985), **syn. nov**. = *Phycosoma japonicum* (Yoshida, 1985). Finally, the first descriptions of the males of *Em. deplanata* (Schenkel, 1936), **comb. nov**. and *Phycosoma ripa* (Zhu, 1998) are provided.

## Introduction

The subfamily Hadrotarsinae Thorell, 1881 is a distinctive group within the family Theridiidae Sundevall, 1833. Many spiders in this subfamily are characterized by a high male carapace (except, e.g., *Anatea* Berland, 1927), sometimes distinctly cylindrical; the presence of two pairs of spermathecae (except, e.g., *Anatea*, *Guaraniella* Baert, 1984, *Tomoxena* Simon, 1895); and short and thin chelicerae bearing long slender fangs for specialized predation on ants (Levi and Levi 1962; Baert 1984; Agnarsson 2004; Cushing 2012; Liu et al. 2016; Smith et al. 2017). Currently, Hadrotarsinae comprises 15 extant genera and 362 species worldwide, of which eight genera and 67 species are recorded from China (Levi 1954; Yoshida 2002; Fitzgerald and Sirvid 2003; Agnarsson 2004; Wunderlich 2008; Lin et al. 2024; Liu et al. 2025; WSC 2026). As pointed out by Agnarsson (2004: 480), the taxonomy of Hadrotarsinae is problematic, with many genera being poorly defined. This paper addresses some of these taxonomic issues.

*Euryopis* Menge, 1868 is the second most diverse genus of the subfamily, with 82 species worldwide (WSC 2026). Levi (1954) considered the genus *Emertonella* Bryant, 1945 as a junior synonym of *Euryopis*, and divided *Euryopis* into two groups based on the male palpal morphology: the emertoni group and the *flavomaculata* group. Yoshida (2002: 17) revalidated *Emertonella*, stating that ‘all the species of the *E. emertoni* group designed by Levi (1954) belong to this genus’. However, he only explicitly transferred *Eu. taczanowskii* Keyserling, 1886, while other species of the group are still treated as *Euryopis* in the WSC (2026). Gao and Li (2014) described two *Emertonella* species from China, and the genus currently comprises four species.

*Phycosoma* O. Pickard-Cambridge, 1880 comprises 32 extant species worldwide (WSC 2026). China represents the diverse centre of *Phycosoma*, with 21 species currently recorded. However, seven of these species are known only from a single sex.

During the examination of Hadrotarsinae specimens collected from China, females of *Phycosoma ripa* (Zhu, 1998) and *Eu. deplanata* Schenkel, 1936 were identified together with their previously unknown males. After examining the male palp of *Em. taczanowskii*, we observed a small cymbial projection that was not reported by Yoshida (2002). This previously unrecognized characteristic contributes to a more precise delimitation of *Emertonella* (Bryant 1945). In the current paper we complete Yoshida’s transfer of the emertoni group species from *Euryopis* to *Emertonella* and provide a revised diagnosis for *Emertonella*; transfer two additional misplaced species and propose a new synonym; and provide the first descriptions of the males of *Phycosoma ripa* and *Eu. deplanata*.

## Materials and methods

The specimens examined in this study were deposited in the Centre for Behavioral Ecology and Evolution (**CBEE**), School of Life Sciences, Hubei University in Wuhan (curator Jie Liu). Specimens were examined using OLYMPUS SZX7 stereomicroscope. Photographs were taken with an OLYMPUS BX51 microscope. All morphological measurements were calculated using a LEICA M205 C stereomicroscope. All measurements were in millimetres (mm). Eyes diameters were taken at the widest point. Legs measurements were given as total length (femur, patella, tibia, metatarsus, tarsus). The male palp was examined and photographed after dissection. The epigyne was dissected from the spider’s body, treated in a warmed 0.1 mg/ml Protease K solution, and stained with Amido black 10B. Terminology follows Agnarsson et al. (2007) and Zhang and Zhang (2012).

**A** atrium;

**ALE** anterior lateral eyes;

**AME** anterior median eyes;

**AS** anterior spermatheca;

**C** conductor;

**CD** copulatory duct;

**CH** cymbial hook;

**CO** copulatory opening;

**CP** cymbial projection;

**EB** embolic base;

**EP** embolic projection;

**ES** embolic spire;

**FD** fertilization duct;

**MA** median apophysis;

**MAH** median apophysis hood;

**PLE** posterior lateral eyes;

**PME** posterior median eyes;

**PS** posterior spermatheca;

**SC** scapus;

**SCD** subcopulatory duct;

**SD** sperm duct;

**Se** septum;

**ST** subtegulum;

**T** tegulum;

**Tb** trichobothri­um;

**TO** tarsal organ;

**TTA** theridiid tegular apophysis;

**I, II, III, IV** legs I to IV.

## Taxonomy

### Family Theridiidae Sundevall, 1833

**Subfamily Hadrotarsinae Thorell, 1881**

#### Genus Emertonella

Taxon classificationAnimaliaAraneaeTheridiidae

Bryant, 1945

E752C13B-23F5-5DC6-A1FB-6A00ACAE2504

##### Type species.

*Emertonella emertoni* (Bryant, 1933) from USA.

##### Diagnosis.

*Emertonella* is similar to *Euryopis* in the absence of conductor, but can be distinguished from *Euryopis* by the male palpal cymbium without projection or with a small lamellar projection (vs cymbium with a wide projection, almost half as wide as cymbium); for females, the two genera are difficult to distinguish.

##### Distribution.

Africa, Asia, Europe, North and South America, and New Guinea.

##### Comments.

Yoshida (2002) proposed that the distinction between *Emertonella* and *Euryopis* lies in the absence of the cymbial projection. However, *Em. deplanata* (Schenkel, 1936), comb. nov. and *Em. taczanowskii* (Keyserling, 1886) exhibit a small lamellar cymbial projection. Accordingly, we re-define *Emertonella* as above.

Yoshida (2002) suggested that species of the emertoni group should be placed in *Emertonella*; however, no formal taxonomic transfers were made (Levi 1954). Herein, 14 species of the emertoni group are formally transferred from *Euryopis* to *Emertonella*: *Em. californica* (Banks, 1904), comb. nov., *Em. coki* (Levi, 1954), comb. nov., *Em. formosa* (Banks, 1908), comb. nov., *Em. funebris* (Hentz, 1850), comb. nov., *Em. lineatipes* (O. Pickard-Cambridge, 1893), comb. nov., *Em. mulaiki* (Levi, 1954), comb. nov., *Em. pepini* (Levi, 1954), comb. nov., *Em. quinquemaculata* (Banks, 1900), comb. nov., *Em. scriptipes* (Banks, 1908), comb. nov., *Em. spinigera* (O. Pickard-Cambridge, 1895), comb. nov., *Em. spiritus* (Levi, 1954), comb. nov., *Em. tavara* (Levi, 1954), comb. nov., *Em. texana* (Banks, 1908), comb. nov., and *Em. varis* (Levi, 1963), comb. nov.

In addition, based on original illustrations and descriptions that conform to the delimitation of *Emertonella*, 16 species previously placed in *Euryopis* are herein transferred to *Emertonella*: *Em. boliviensis* (Rodrigues, Marta & Figueiredo, 2021), comb. nov., *Em. camis* (Levi, 1963), comb. nov., *Em. catarinensis* (Rodrigues, Marta & Figueiredo, 2021), comb. nov., *Em. cobreensis* (Levi, 1963), comb. nov., *Em. cyclosisa* (Zhu & Song, 1997), comb. nov., *Em. emiliae* (Lecigne, 2023), comb. nov., *Em. episinoides* (Walckenaer, 1847), comb. nov., *Em. mallah* (Zakerzade, Moradmand & Jäger, 2022), comb. nov., *Em. nasuta* (Rodrigues, Marta & Figueiredo, 2021), comb. nov., *Em. nigra* (Yoshida, 2000), comb. nov., *Em. pickardi* (Levi, 1963), comb. nov., *Em. quinqueguttata* (Thorell, 1875), comb. nov., *Em. sexmaculata* (Hu, 2001), comb. nov., *Em. spinifera* (Mello-Leitão, 1944), comb. nov., *Em. talaveraensis* (González, 1991), comb. nov., and *Em. weesei* (Levi, 1963), comb. nov. (Levi 1963; González 1990, 1991; Heimer and Nentwig 1991; Zhu 1998; Yoshida 2000; Hu 2001; Chen et al. 2017; Rodrigues et al. 2021; Zakerzade et al. 2022; Lecigne 2023). However, due to unclear illustrations (e.g., *Eu. elegans* Keyserling, 1890), or misplacements (e.g., *Eu. galeiforma* Zhu, 1998, belonging to Theridiinae, Liu et al. 2025), sufficient information is lacking to confidently determine their generic placement (Hickman 1967; Rodrigues et al. 2021; Shi et al. 2025). These species are therefore retained in their current placements.

*Euryopis mingyaoi* Yin, 2012 fits the diagnostic characteristics of *Physcoa* Thorell, 1895 in possessing a prolateral-distal cymbial projection and a retrolaterally positioned paracymbium (cymbial hood). It is similar to *Physcoa oxycera* (Zhu & Song, 1993) in the shape of the median apophysis, conductor, embolus, and overall habitus colouration. In addition, the presence of two promarginal cheliceral teeth (cf. Yin et al. 2012: fig. 142a–f and Hu et al. 2026: figs 8A–D, 13A, G) indicates this species does not belong to Hadrotarsinae. However, as the holotype of this species appears to be lost, we were unable to study its detailed morphology. Nevertheless, based on the available evidence, we transfer *Eu. mingyaoi* to *Physcoa* as a new combination, *Physcoa mingyaoi* (Yin, 2012), comb. nov.

#### Emertonella deplanata

Taxon classificationAnimaliaAraneaeTheridiidae

(Schenkel, 1936) comb. nov.

BF895997-62D1-585E-AA4B-1E309D2AA38F

**Figure 1.**
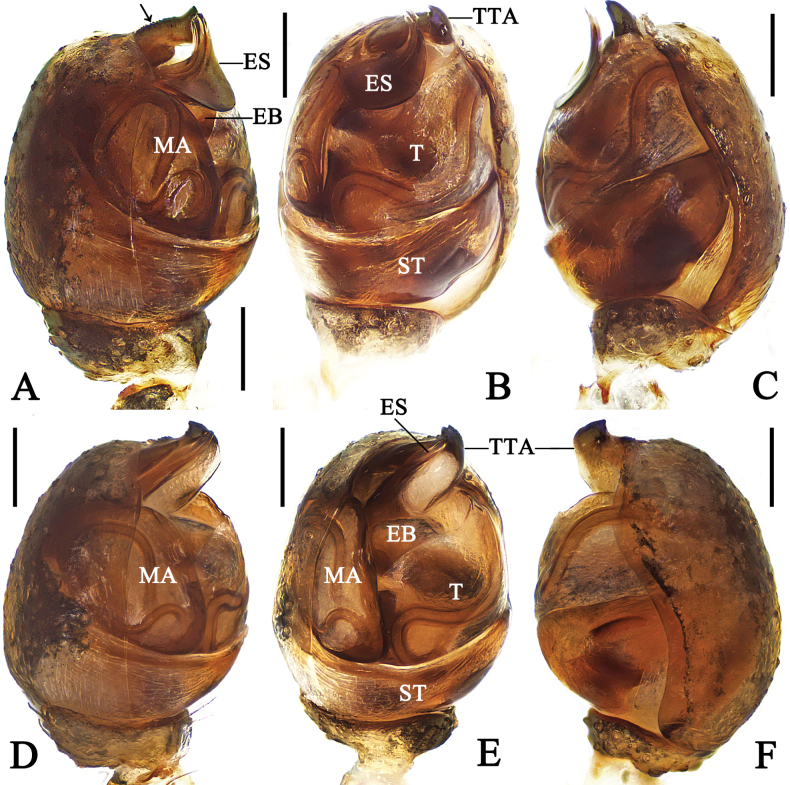
Left male palp of *Emertonella* spp. **A–C**. *Em. deplanata* (Schenkel, 1936), comb. nov. (LJ202001623); **D–F**. *Em. taczanowskii* (Keyserling, 1886) (LJ201903893); **A, D**. Prolateral view (arrow in **A**. Points to the denticles); **B, E**. Ventral view; **C, F**. Retrolateral view. Abbreviations: EB–embolic base; ES–embolic spire; MA–median apophysis; ST–subtegulum; T–tegulum; TTA–theridiid tegular apophysis. Scale bars: 0.1 mm. (Photographs by FL).

**Figure 2.**
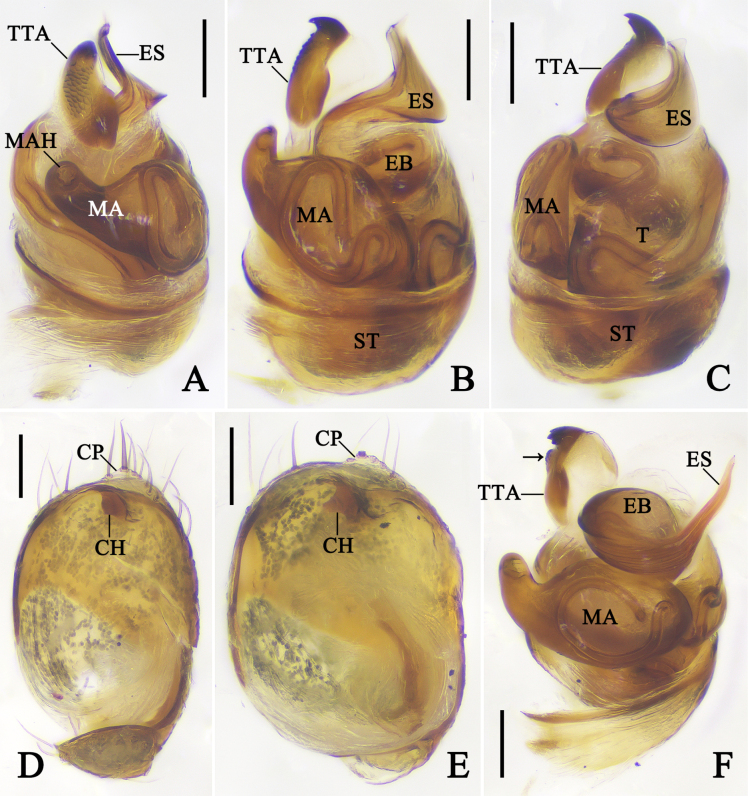
Expanded right male palp of *Emertonella* spp., flip horizontal. **A–D**. *Em. deplanata* (Schenkel, 1936), comb. nov. (LJ202001623); **E, F**. *Em. taczanowskii* (Keyserling, 1886) (LJ201903893); **A**. Bulb, dorsal view; **B, F**. Bulb, prolateral view (arrow in **F**. Points to the hump); **C**. Bulb, ventral view; **D, E**. Cymbium, ventral view. Abbreviations: CH–cymbial hook; CP–cymbial projection; EB–embolic base; ES–embolic spire; MA–median apophysis; ST–subtegulum; T–tegulum; TTA–theridiid tegular apophysis. Scale bars: 0.1 mm. (Photographs by CH).

**Figure 3.**
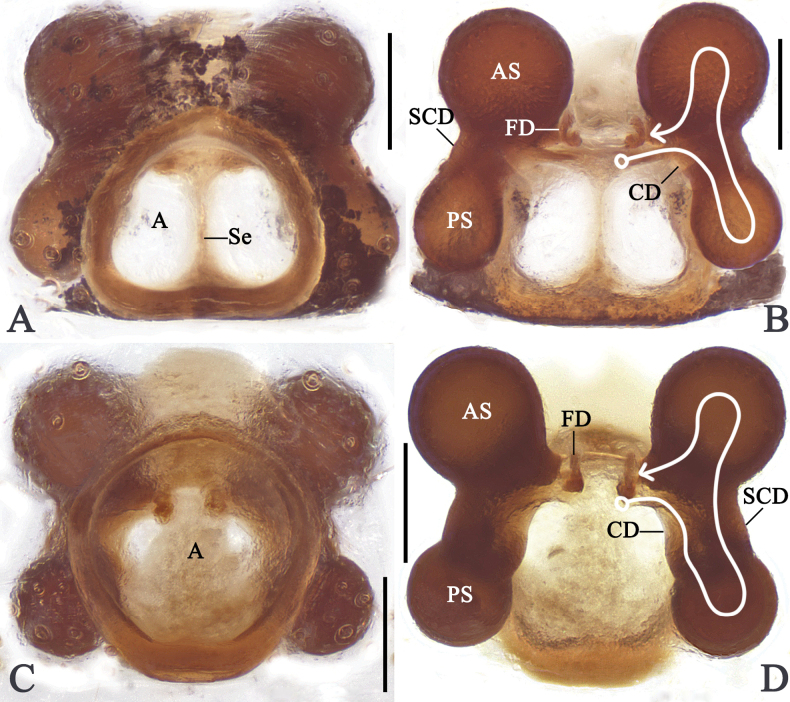
Female genitalia of *Emertonella* spp. **A, B**. *Em. deplanata* (Schenkel, 1936), comb. nov. (LJ202001798); **C, D**. *Em. taczanowskii* (Keyserling, 1886) (LJ201903320); **A, C**. Epigyne, ventral view; **B, D**. Vulva, dorsal view. Abbreviations: A–atrium; AS–anterior spermatheca; CD–copulatory duct; FD–fertilization duct; PS–posterior spermatheca; SCD–subcopulatory duct; Se–septum. Scale bars: 0.1 mm. (Photographs by FL).

**Figure 4.**
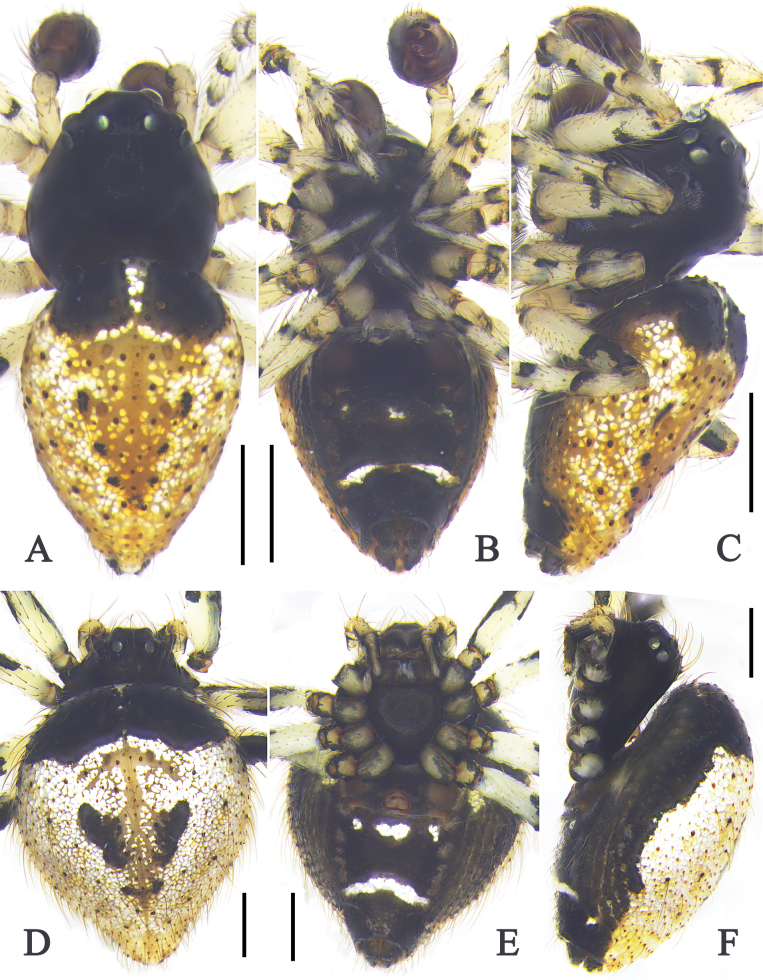
Habitus of *Emertonella deplanata* (Schenkel, 1936), comb. nov. **A–C**. Male (LJ202001623); **D–F**. Female (LJ202001798); **A, D**. Dorsal view; **B, E**. Ventral view; **C, F**. Lateral view. Scale bars: 0.5 mm. (Photographs by FL).

Figs 1A–C, 2A–D, 3A, 3B, 4

Euryopis deplanata Schenkel, 1936: 45, fig. 13 (holotype: female, China, Sichuan Province, Hsin-lung-chang; elev. 450 m; 11 May 1930; Hummel leg.; depository institution unknown; not examined); Song et al. 1999: 123 (may be a synonym of Em. taczanowskii, rejected here).
Euryopis taczanowskii Zhu, 1998: 37, fig. 17A–C (female, misidentified); Song et al. 1999: 123, fig. 63C, D (female, misidentified).
Emertonella taczanowskii Yin et al., 2012: 329, fig. 130a–e (female, misidentified); Rajoria 2015: 46, figs 5–8 (female, misidentified).
Emertinella taczanowskii Khan et al., 2025: 135, figs 14, 15 (female, misidentified).

##### Material examined.

China • 1 male (LJ202001181); Henan Province, Xinyang City, Xin County, Liankangshan, Laomiao Village; 31.6528°N, 114.7861°E; elev. 230 m; 10 July 2020; Fengjie Liu leg. • 2 males, 1 female (LJ202001603, 202001623, 202001629); Henan Province, Xinyang City, Jingangtai, Donghe Village; 31.7500°N, 115.5550°E; elev. 480 m; 19 July 2020; Fengjie Liu leg. • 1 female (LJ202001744); Henan Province, Xinyang City, Shangcheng County, Fengji Town, Huangwan; 31.8703°N, 115.5331°E; elev. 120 m; 20 July 2020; Fengjie Liu leg. • 1 male, 2 females (LJ202001787, 202001798, 202001857); Henan Province, Xinyang City, Huangchuan County, Sili Village; 31.9506°N, 115.1028°E; elev. 90 m; 22 July 2020; Fengjie Liu leg. • 1 male (LJ202001826); Henan Province, Xinyang City, Guangshan County, Luochen Town, Qianchengwan; 31.9600°N, 114.6803°E; elev. 70 m; 22 July 2020; Fengjie Liu leg. • 2 females (LJ202001855, 202001873); Henan Province, Xinyang City, Luoshan County, Luzhai Village; 31.9158°N, 114.4967°E; elev. 90 m; 22 July 2020; Fengjie Liu leg. • 1 female (LJ202001908); Henan Province, Nanyang City, Tongbai County; 32.3192°N, 113.3517°E; elev. 280 m; 23 July 2020; Fengjie Liu leg. • 1 female (LJ202002090); Henan Province, Nanyang City, Tongbai County, Huaiyuan Village; 32.4161°N, 113.2744°E; elev. 260 m; 26 July 2020; Fengjie Liu leg. • 3 females (LJ202002127, 202002155, 202002158); Henan Province, Nanyang City, Tongbai County, Dujuanyuan; elev. 810 m; 27 July 2020; Fengjie Liu leg. • 1 male (LJ202002184); Henan Province, Nanyang City, Baotianman, Pingfang; elev. 1340 m; 28 July 2020; Fengjie Liu leg.

##### Diagnosis.

**Figure 5.**
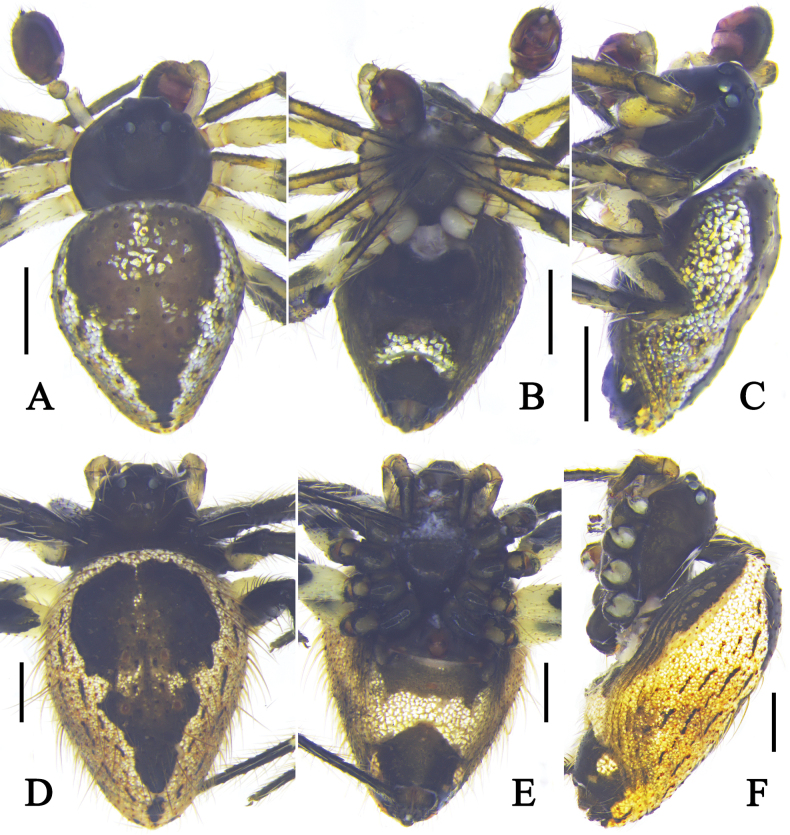
Habitus of *Emertonella taczanowskii* (Keyserling, 1886). **A–C**. Male (LJ201903893); **D–F**. Female (LJ201903320); **A, D**. Dorsal view; **B, E**. Ventral view; **C, F**. Lateral view. Scale bars: 0.5 mm. (Photographs by FL).

*Emertonella deplanata* (Schenkel, 1936), comb. nov. is similar to Chinese *Em. taczanowskii* (cf. Figs 1A–C, 2A–D, 3A, 3B, 4and Figs 1D–F, 2E, 2F, 3C, 3D, 5), but can be distinguished by: 1. dorsal opisthosoma with two black anterior markings (vs with a black triangular marking), 2. embolic spire broad and curved (vs needle like and straight), 3. atrium triangular (vs rounded), and 4. interdistance between anterior spermathecae obviously shorter than that between the posterior pair (vs almost as long as that between the posterior pair).

##### Description.

**Male** (LJ202001623): Total length 2.00. Carapace 0.68 long, 0.67 wide. Opisthosoma 1.16 long, 0.88 wide. Eyes: AME 0.08, ALE 0.05, PME 0.05, PLE 0.05, AME–AME 0.15, AME–ALE 0.09, PME–PME 0.12, PME–PLE 0.12, AME–PME 0.10, ALE–PLE 0.02. Measurements of legs: I 2.05 (0.62, 0.21, 0.48, 0.40, 0.34), II 2.09 (0.60, 0.20, 0.45, 0.64, 0.20), III 1.82 (0.58, 0.16, 0.37, 0.50, 0.21), IV 2.27 (0.65, 0.21, 0.52, 0.62, 0.27). Leg formula: IV-II-I-III.

***Palp*** (Figs 1A–C, 2A–D): Tibia ~ 1/4 length of cymbium. Cymbium oval, with a small lamellar projection. Cymbial hook triangular, situated anterior margin of alveolus. Subtegulum bowl like. Tegulum quadrangular. Median apophysis triangular in prolateral view. Theridiid tegular apophysis sclerotized, anterior margin with denticles (arrow in Fig. 1A) and distal part with two tiny denticles. Embolic spire semicircular, broad, and directed anticlockwise, arising dorsally from embolic base.

***Colouration*** (Fig. 4A–C): Carapace, sternum, endites and labium black. Palps and legs yellow with black markings and rings. Dorsal opisthosoma yellow, with white patches and black flecks, anteriorly with two black markings; venter black, posterior part of genital groove with three white spots, anterior part of spinnerets with a white line. Spinnerets black.

**Female** (LJ202001798): Total length 2.64. Carapace 0.89 long, 0.86 wide. Opisthosoma 1.89 long, 1.47 wide. Eyes: AME 0.08, ALE 0.07, PME 0.07, PLE 0.07, AME–AME 0.12, AME–ALE 0.05, PME–PME 0.12, PME–PLE 0.06, AME–PME 0.10, ALE–PLE 0.05. Measurements of legs: I 2.57 (0.77, 0.29, 0.57, 0.58, 0.36), II 2.69 (0.66, 0.25, 0.72, 0.66, 0.40), III 2.48 (0.68, 0.30, 0.49, 0.59, 0.42), IV 3.10 (0.95, 0.24, 0.52, 0.87, 0.52). Leg formula: IV-II-I-III.

***Epigyne*** (Fig. 3A, B): Epigyne with a triangular atrium, with slight septum; anterior margins of atrium as thick as posterior margins. Copulatory openings unobvious, situated anterior atrium. Copulatory ducts and subcopulatory ducts fused and unobvious. Posterior spermathecae spherical. Anterior spermathecae spherical, ~ 1.5× wider than posterior pair. Fertilization ducts curved, arising from posterior part of anterior spermathecae.

***Colouration*** (Fig. 4D–F): As in male, but generally lighter. Median part of dorsal opisthosoma with a V-shaped black marking.

##### Distribution.

China (Henan, Hunan, Sichuan, Yunnan), India, Pakistan.

##### Comments.

This species was originally described based on a female specimen collected from Sichuan Province, China, with only illustrations of the opisthosoma and ventral epigyne provided (Schenkel 1936). The presence of two black markings on the anterior dorsal opisthosoma and the triangular epigynal atrium clearly supports the accuracy of our identification (cf. Figs 3A, 4D, 4E and Schenkel 1936: fig. 13). The congruent opisthosomal pattern and the fact that males and females were collected together confirm the correct sex association. In addition, the male palp of this species exhibits the diagnostic characteristic of *Emertonella* as defined above. Therefore, we transfer this species from *Euryopis* to *Emertonella* as a new combination.

#### Emertonella taczanowskii

Taxon classificationAnimaliaAraneaeTheridiidae

(Keyserling, 1886)

1BC710A1-C608-5228-8D90-CA18D95C88C4

Figs 1D–F, 2E, 2F, 3C, 3D, 5

Euryopis taczanowskii Keyserling, 1886: 47, pl. 12, fig. 160 (holotype: female, PERU, Tumbes; deposited in Polish Academy of Sciences; not examined); Levi 1963: 132, fig. 10 (synonym of Eu. floricola, Eu. nigripes and Eu. rosascostai); Levi 1967: 178, figs 37–41 (male and female); Yoshida 1992: 139, figs 1–4 (male); Zhu 1998: 37, fig. 17D (male, republication of the original figure); Song et al. 1999: 123, fig. 63G (male, republication of the original figure).
Euryopis floricola Keyserling, 1886: 261, pl. 21, fig. 309 (female).
Euryopis nigripes Banks, 1929: 86, pl. 3, fig. 47, pl. 4, fig. 60 (female); Levi 1954: 24, figs 38–52 (male and female, synonym of Eu. dentata).
Euryopis dentata Gertsch & Mulaik,1936: 6, figs 10, 11 (male and female).
Euryopis rosascostai Mello-Leitão, 1944: 325, fig. 6 (female).
Emertonella taczanowskii Yoshida, 2002: 17, figs 31–34 (male and female, transfer from Euryopis); Yoshida 2003: 187, figs 524–527 (male and female); Yoshida 2009: 393, figs 357–359 (male and female); Yin et al. 2012: 329, fig. 130f (male, republication of the original figure); Almaraz-López et al. 2025: 139, fig. 5a, b, l (female).

##### Material examined.

China • 1 male (LJ201903893); Yunnan Province, Xishuangbanna Dai Autonomous Prefecture, Xishuangbanna Botanical Garden, Tropical Rainforest; 21.9281°N, 101.2558°E; elev. 550 m; 22 September 2019; Jian Chen et al. leg. • 1 female (LJ201903320); Yunnan Province, Puer City, Ximahe Park; 22.7897°N, 100.9842°E; elev. 1340 m; 19 September 2019; Jian Chen et al. leg. • 1 female (LJ202003300); Yunnan Province, Xishuangbanna Dai Autonomous Prefecture, Xishuangbanna Botanical Garden; 21 July 2020; collector unknown.

##### Diagnosis.

*Emertonella taczanowskii* is similar to *Em. nasuta* (Rodrigues, Marta & Figueiredo, 2021), comb. nov. in having a needle like embolic spire pointing to 1:30 o’clock position (cf. Figs 1D–F, 3C, 3D, 5 and Rodrigues et al. 2021: figs 3, 4, 8B, 9C, D), but can be distinguished by: 1. opisthosoma triangular (vs oval), 2. theridiid tegular apophysis ox horn shaped in ventral view (vs triangular), 3. atrium larger than spermathecae (vs smaller than spermathecae), and 4. anterior spermathecae larger than the posterior pair (vs smaller than the posterior pair).

##### Redescription.

**Male** (LJ201903893): Total length 1.93. Carapace 0.72 long, 0.67 wide. Opisthosoma 1.25 long, 1.05 wide. Eyes: AME 0.05, ALE 0.06, PME 0.05, PLE 0.06, AME–AME 0.13, AME–ALE 0.09, PME–PME 0.12, PME–PLE 0.08, AME–PME 0.11, ALE–PLE 0.02. Measurements of legs: I 1.73 (0.57, 0.22, 0.45, 0.27, 0.22), II 2.03 (0.52, 0.27, 0.45, 0.50, 0.29), III 1.88 (0.59, 0.19, 0.38, 0.48, 0.24), IV 2.63 (0.80, 0.26, 0.55, 0.64, 0.38). Leg formula: IV-II-III-I.

***Palp*** (Figs 1D–F, 2E, 2F): Tibia ~ 1/5 length of cymbium. Cymbium oval, with a small lamellar projection. Cymbial hook triangular, situated anterior margin of alveolus. Subtegulum bowl-like. Tegulum quadrangular. Median apophysis triangular in prolateral view. Theridiid tegular apophysis sclerotized, dorsally with a hump and denticles (arrow in Fig. 2F), distal part with three denticles. Embolic base swollen; embolic spire needle like.

***Colouration*** (Fig. 5A–C): Carapace and sternum brownish-black. Legs yellow to brown. Dorsal opisthosoma with white patches, medially with a triangular black marking, laterally with black spots; venter dark brown, anterior part of spinnerets with a white line. Spinnerets brown.

**Female** (LJ201903320): Total length 3.02. Carapace 0.92 long, 0.92 wide. Opisthosoma 2.01 long, 1.68 wide. Eyes: AME 0.10, ALE 0.09, PME 0.08, PLE 0.07, AME–AME 0.12, AME–ALE 0.10, PME–PME 0.16, PME–PLE 0.12, AME–PME 0.13, ALE–PLE 0.04. Measurements of legs: I 3.15 (0.84, 0.35, 0.67, 0.84, 0.45), II 3.47 (0.89, 0.37, 0.68, 0.97, 0.56), III 3.33 (0.98, 0.31, 0.71, 0.81, 0.52), IV 4.06 (0.98, 0.37, 0.92, 1.31, 0.48). Leg formula: IV-II-III-I.

***Epigyne*** (Fig. 3C, D): Epigyne with a rounded atrium, anterior margins of atrium thinner than posterior margins. Copulatory openings unobvious, situated anterior atrium. Copulatory ducts and subcopulatory ducts fused and unobvious. Posterior spermathecae spherical. Anterior spermathecae spherical, ~ 1.5× wider than posterior pair. Fertilization ducts curved, arising from posterior part of anterior spermathecae.

***Colouration*** (Fig. 5D–F): as in male, but generally darker.

##### Distribution.

China (Yunnan), Japan, New Guinea, Sri Lanka, USA to Argentina (WSC 2026).

##### Comments.

Levi (1954) considered this species to be polytypic, exhibiting two pattern phases and slight variations in theridiid tegular apophysis and epigyne (e.g., Keyserling 1886: pl. 12 fig. 160, pl. 21 fig. 309, Levi 1954: figs 39–41, 45–48, 50–52; Yoshida 1992: figs 1–4). During the study of publications referring to “*Em. taczanowskii*”, we found that female specimens from China, India, and Pakistan were characterized by a triangular atrium, the interdistance between the anterior spermathecae clearly shorter than that between the posterior pair, and two black markings on the anterior dorsal opisthosoma (fig. Zhu 1998: 17A–C; Song et al. 1999: fig. 63C, D; Yin et al. 2012: fig. 130a, b, d, e; Rajoria 2015: figs 5–8; Khan et al. 2025: figs 14, 15) conform well to *Eu. deplanata* (fig. 13 in Schenkel 1936, transfer to *Emertonella* in the current paper) in the morphology of the epigyne and opisthosoma. Notably, no matching males were examined in these studies. We therefore consider that the specimens reported in the aforementioned publications were misidentified and should be referred to *Em. deplanata* (Schenkel, 1936), comb. nov.

In the current study, specimens collected from Yunnan, China, exhibit an epigyne with a rounded atrium and the interdistance between the anterior spermathecae almost as wide as that between the posterior pair, fully matching the illustrations provided by Levi (Levi 1954: figs 50–52). Accordingly, the identification of our specimens as *Em. taczanowskii* is confirmed.

#### Genus Phycosoma

Taxon classificationAnimaliaAraneaeTheridiidae

O. Pickard-Cambridge, 1880

95DB8C9B-47C1-53B6-B2B0-99A4AF9607DA

##### Type species.

*Phycosoma oecobioides* O. Pickard-Cambridge, 1880 from New Zealand.

##### Diagnosis.

*Phycosoma* can be distinguished from other Hadrotarsinae genera by the following combination of characteristics: 1. male with high carapace (cylindrical in many species) and slightly raised cephalic region, 2. median apophysis large, with S-shaped sperm duct (except *Phycosoma corrugum* Gao & Li, 2014, *Phycosoma ripa* (Zhu, 1998), and *Phycosoma turriceps* (Schenkel, 1936)), 3. embolus with a wide base situated anterior position of tegulum, embolic spire small, 4. theridiid tegular apophysis and conductor small, situated anterior distal part of bulb, 5. posterior part of epigyne with a scape, copulatory opening posteriorly situated (except *Phycosoma turriceps*) (Gao and Li 2014; Liu et al. 2025).

##### Distribution.

Worldwide.

##### Comments.

*Phycosoma stigmosum* Yin, 2012 was originally described based on a male specimen from Hunan, China. The morphology of the palp shows that the embolus lacks a wide base, a characteristic feature of the genus *Lasaeola* Simon, 1881, especially of the *castrata* group (Liu et al. 2025). However, as the holotype of this species appears to be lost, we were unable to study its detailed morphology. Nevertheless, based on the available evidence, we transfer *Phycosoma stigmosum* to *Lasaeola* as a new combination: *L. stigmosum* (Yin, 2012), comb. nov.

Yoshida reported two species from Japan, *Pholcomma japonicum* Yoshida, 1985 and *Pho. amamiense* Yoshida, 1985, and subsequently transferred both species to *Dipoena* Thorell, 1869 (Yoshida 1985, 1991). Paik (1996) described *D. kayaensis* Paik, 1996 from Korea. Yoshida and Ono (2000) considered *D. kayaensis* to be a junior synonym of *D. japonica* (now *Phycosoma japonicum*), and Marusik and Koponen (2000) regarded *D. kayaensis* as a junior synonym of *D. amamiensis* (now *Phycosoma amamiense*). Yoshida and Ono (2000) proposed that the distinction between the two species lies only in habitus length. Basen on morphological comparison, we found that the two species share a same sclerotized plate on the anterior dorsal opisthosoma of the male, the identical sperm duct configuration inside the tegulum, median apophysis, and embolic base, and congruent structures of the embolus and vulva (Yoshida 1985, 1989, 1991, 2002, 2003, 2009; Zhu 1992, 1998; Paik 1995, 1996; Kim and Kim 1997; Song et al. 1999; Marusik and Koponen 2000; Yoshida and Ono 2000; Fitzgerald and Sirvid 2004; Zhu and Zhang 2011; Yin et al. 2012; Kim 2021; Xie et al. 2021; Suzuki et al. 2023, 2025; Hino 2024; Suzuki and Hisasue 2024; Hidaka and Eto 2025). Accordingly, we consider *Phycosoma amamiense* (Yoshida, 1985), syn. nov. to be a junior synonym of *Phycosoma japonicum* (Yoshida, 1985).

#### Phycosoma ripa

Taxon classificationAnimaliaAraneaeTheridiidae

(Zhu, 1998)

44D99A00-103A-5555-B3DB-04295F986537

Figs 6, 7, 8

Dipoena ripa Zhu 1998: 233, 376, fig. 152A–D (holotype: female, CHINA, Hubei Province: Enshi Tujia and Miao Autonomous Prefecture, Hefeng County; 29°48'N, 110°00'E; 30 May 1989; Mingsheng Zhu leg.; deposited in the Museum of Hebei University, Hebei University, examined); Song et al. 1999: 112, fig. 56C, D (female).
Phycosoma ripa Liu et al., 2025: 325 (transfer from Dipoena).

##### Material examined.

China • 1 male, 2 females (QZMS00175, 00706, 00707; Hubei Province, Enshi Tujia and Miao Autonomous Prefecture, Xuan’en County, Shadaogou Town, Longtan Village; 29.6953°N, 109.6628°E; elev. 637 m; 17 July 2023; Changhao Hu and Mian Wei leg. • 1 male, 1 female (QZMS04197, 04198); Enshi Tujia and Miao Autonomous Prefecture, Xuan’en County, Shadaogou Town, Baishuihe Village; 29.9238°N, 109.7360°E; elev. 843 m; 23–24 July 2023; Changhao Hu and Mian Wei leg.

##### Diagnosis.

**Figure 6.**
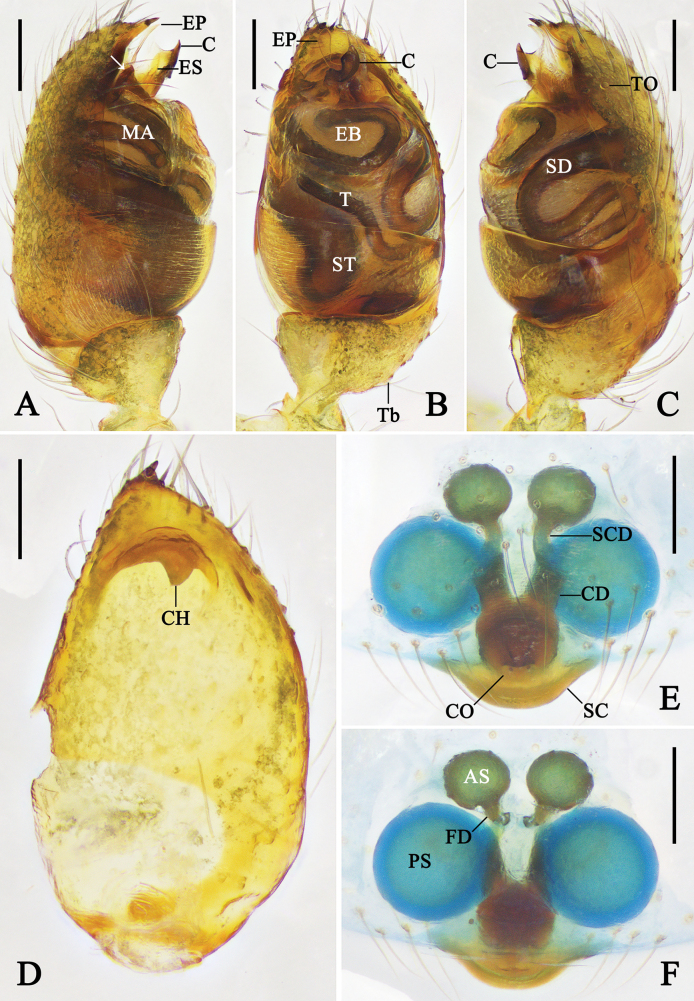
Copulatory organs of *Phycosoma ripa* (Zhu, 1998). **A–D**. Left male palp (QZMS00175); **E, F**. Female genitalia (QZMS00706); **A**. Prolateral view (arrow points to the projection); **B**. Ventral view; **C**. Retrolateral view; **D**. Cymbium, ventral view; **E**. Epigyne, ventral view; **F**. Vulva, dorsal view. Abbreviations: AS–anterior spermatheca; C–conductor; CD–copulatory duct; CH–cymbial hook; CO–copulatory opening; EB–embolic base; EP–embolic projection; ES–embolic spire; FD–fertilization duct; MA–median apophysis; PS–posterior spermatheca; SC–scapus; SCD–subcopulatory duct; SD–sperm duct; ST–subtegulum; T–tegulum; Tb–trichobothrium; TO–tarsal organ. Scale bars: 0.1 mm. (Photographs by CH).

**Figure 7.**
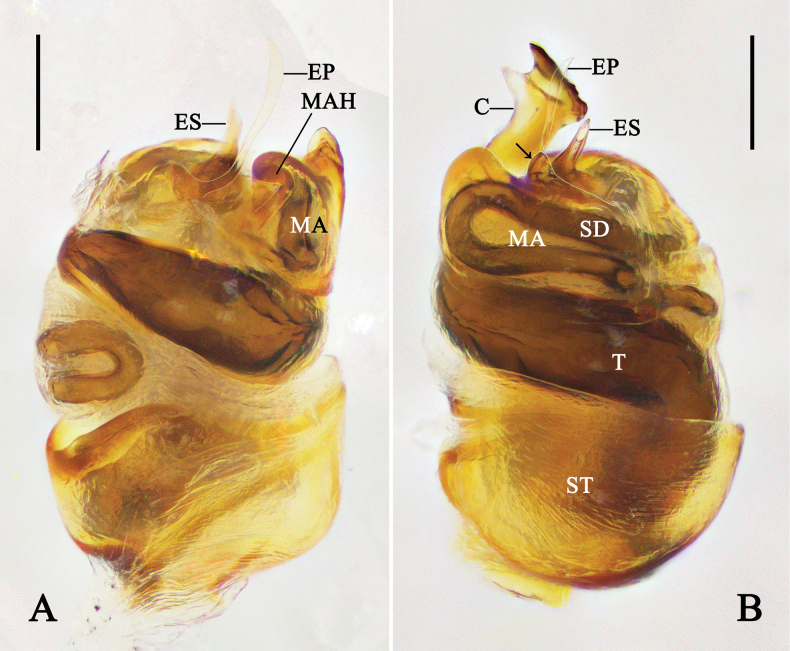
Expanded bulb of left male palp of *Phycosoma ripa* (Zhu, 1998) (QZMS00175). **A**. Dorsal view, removed conductor; **B**. Prolateral view (arrow points to the projection). Abbreviations: C–conductor; EP–embolic projection; ES–embolic spire; MA–median apophysis; MAH–median apophysis hood; SD–sperm duct; ST–subtegulum; T–tegulum. Scale bars: 0.1 mm. (Photographs by CH).

The male of *Phycosoma ripa* is similar to *Phycosoma corrugum* Gao & Li, 2014 (cf. Figs 6A–C, 7 and Gao and Li 2014: figs 51A, B, 52, 54) in having a parallel sperm duct inside median apophysis, and curved conductor in ventral view, but can be distinguished by: 1. sperm duct inside retrolateral part of tegulum S-shaped (vs U-shaped), and 2. embolic base and sperm duct almost triangular (vs quadrangular). The female of *Phycosoma ripa* is similar to *Phycosoma hainanense* (Zhu, 1998) (cf. Fig. 6E, F and Zhu 1998: fig. 153B, C) in having a labiate scapus, rounded sclerotized part around copulatory opening, and the copulatory duct connecting to interior part of spermatheca, but can be distinguished by: 1. the whole carapace brownish-green (vs lateral part of carapace yellow, median part brownish-black), 2. scapus ~ 3× wider than long (vs ~ 1.5× wider than long), and 3. copulatory opening heart-shaped (vs rounded).

##### Description.

**Male** (QZMS00175): Total length 1.73. Carapace 0.89 long, 0.78 wide. Opisthosoma 0.92 long, 0.70 wide. Eyes: AME 0.08, ALE 0.05, PME 0.07, PLE 0.06, AME–AME 0.10, AME–ALE 0.02, PME–PME 0.06, PME–PLE 0.07, AME–PME 0.04, ALE–PLE 0.00. Measurements of legs: I 2.22 (0.72, 0.24, 0.51, 0.44, 0.31), II 1.96 (0.63, 0.21, 0.43, 0.39, 0.30), III 1.85 (0.59, 0.20, 0.39, 0.35, 0.32), IV 2.25 (0.68, 0.23, 0.57, 0.44, 0.33). Leg formula: IV-I-II-III.

***Palp*** (Figs 6A–D, 7): Tibia ~ 1/3 length of cymbium, with distal part ~ 4× wider than proximal part in ventral view; retrolateral part with a trichobothrium. Cymbium oval, distally with a short spine; tarsal organ ~ 2× larger than setal sockets; cymbial hook almost triangular. Subtegulum bowl-like. Tegulum retrolaterally with S-shaped sperm duct. Median apophysis apically with a projection (arrows in Figs 6A, 7B), sperm duct parallel inside median apophysis; median apophysis hood triangular. Conductor curved in ventral view, dorsally slightly sclerotized and ventrally strongly sclerotized. Embolic base almost triangular, with sperm duct looping triangularly; embolic projection long knife like, ~ 2/5 the length of bulb; embolic spire needle like, almost half the length of embolic projection.

**Figure 8.**
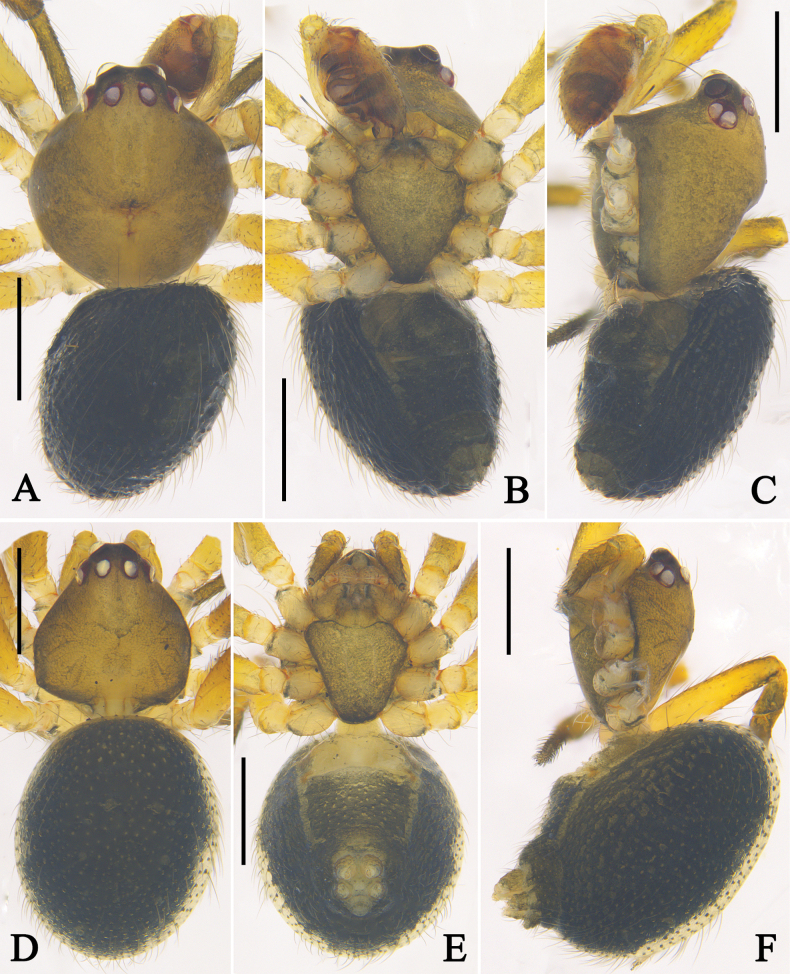
Habitus of *Phycosoma ripa* (Zhu, 1998). **A–C**. Male (QZMS00175); **D–F**. Female (QZMS00706); **A, D**. Dorsal view; **B, E**. Ventral view; **C, F**. Lateral view. Scale bars: 0.5 mm. (Photographs by CH).

***Colouration*** (Fig. 8A–C): Carapace and sternum brownish-green, with black margins. Chelicerae pale brown. Endites brownish-green. Legs orange to dark brown. Opisthosoma black. Spinnerets dark brown.

**Female** (QZMS00706): Total length 1.90. Carapace 0.74 long, 0.75 wide. Opisthosoma 1.09 long, 0.96 wide. Eyes: AME 0.09, ALE 0.06, PME 0.08, PLE 0.06, AME–AME 0.07, AME–ALE 0.01, PME–PME 0.06, PME–PLE 0.06, AME–PME 0.06, ALE–PLE 0.02. Measurements of legs: I 2.04 (0.66, 0.21, 0.46, 0.41, 0.30), II 2.02 (0.67, 0.23, 0.46, 0.34, 0.32), III 1.89 (0.59, 0.23, 0.37, 0.39, 0.31), IV 2.37 (0.73, 0.27, 0.55, 0.47, 0.35). Leg formula: IV-I-II-III.

***Epigyne*** (Fig. 6E, F): Epigyne posteriorly with a labiate scapus. Copulatory opening heart-shaped, with a rounded sclerotized part. Copulatory ducts straight, almost half the width of the sclerotized part and almost as long as the sclerotized part. Posterior spermathecae spherical, membranous. Subcopulatory ducts slightly curved, almost as long as copulatory ducts and almost half the width of the copulatory ducts. Anterior spermathecae spherical, almost half the width of posterior spermathecae. Fertilization duct curved, arising from posterior part of anterior spermathecae.

***Colouration*** (Fig. 8D–F) as in male, but generally paler.

##### Distribution.

China (Hubei).

##### Comments.

After comparison between our female specimens with the holotype of *Phycosoma ripa*, no differences were found, indicating that our identification is correct. The congruent habitus colouration and the fact that males and females were collected together confirm the correct sex association.

## Discussion

The subfamily Hadrotarsinae is a worldwide group with a long taxonomic history. Yoshida (2002) made a significant contribution to the taxonomy of Hadrotarsinae spiders from eastern Asia. Liu et al. (2025) provided the first molecular phylogeny of eastern Asian Hadrotarsinae and resolved several longstanding taxonomic issues. However, the lack of a global background means that numerous problems within this subfamily remain unresolved. In the current paper, we transfer *Eu. deplanata* and 30 additional *Euryopis* species to *Emertonella*, *Eu. mingyaoi* Yin, 2012 to *Physcoa*, and *Phycosoma stigmosum* Yin, 2012 to *Lasaeola* based on morphological evidence. We also provide the first descriptions of the males of *Phycosoma ripa* and *Em. deplanata* (Schenkel 1936), comb. nov., and propose a new synonym, *Phycosoma amamiense* (Yoshida, 1985), syn. nov. = *Phycosoma japonicum* (Yoshida, 1985).

We found that there are some potential synonyms within the genus *Phycosoma*, for example between *Phycosoma nigromaculatum* (Yoshida, 1987) and *Phycosoma stellare* (Zhu, 1998) (Zhu 1998; Yoshida 2009; Zhang and Zhang 2012). Both species possess a sclerotized plate on the male dorsal opisthosoma and small flecks on the female dorsal opisthosoma, and they share similar structures of sperm duct inside the tegulum, median apophysis, and embolic base. Unfortunately, the types of these species are unavailable for examination, and the published illustrations are insufficient to clearly show the structure of copulatory ducts.

While we examined specimens from China, we discovered that numerous specimens exhibit transitional epigynal morphologies, among them *Phycosoma mustelinum* (Simon, 1889), *Phycosoma sinicum* (Zhu, 1992), and *Phycosoma submustelinum* (Zhu, 1998). This phenomenon suggests that delimitation of these three species requires further study.

In summary, substantial taxonomic issues remain within the Hadrotarsinae, likely owing to the small body size of these spiders, the complex morphology of male palp, and the relatively simple structure of female genitalia. We suggest that future taxonomic studies of Hadrotarsinae, and of Theridiidae more broadly, should provide expanded and detailed illustrations of the male palp to clearly demonstrate sclerites and facilitate more robust species identification.

## Supplementary Material

XML Treatment for Emertonella

XML Treatment for Emertonella deplanata

XML Treatment for Emertonella taczanowskii

XML Treatment for Phycosoma

XML Treatment for Phycosoma ripa

## Additional information

### Use of AI

No use of AI was reported.

### Author ORCIDs

Changhao Hu https://orcid.org/0009-0007-5591-3121

He Zhang https://orcid.org/0000-0002-1478-9837

Fengjie Liu https://orcid.org/0000-0002-7727-0493

### Data availability

All of the data that support the findings of this study are available in the main text.
